# Current trends suggest most Asian countries are unlikely to meet future biodiversity targets on protected areas

**DOI:** 10.1038/s42003-022-04061-w

**Published:** 2022-11-29

**Authors:** Mohammad S. Farhadinia, Anthony Waldron, Żaneta Kaszta, Ehab Eid, Alice Hughes, Hüseyin Ambarlı, Hadi Al- Hikmani, Bayarbaatar Buuveibaatar, Mariya A. Gritsina, Iding Haidir, Zafar-ul Islam, Muhammad Kabir, Gopal Khanal, Maxim A. Koshkin, Rahim Kulenbekov, Zairbek Kubanychbekov, Aishwarya Maheshwari, Ugyen Penjor, Hana Raza, Tatjana Rosen, Anna Yachmennikova, Viatcheslav V. Rozhnov, Nobuyuki Yamaguchi, Paul J. Johnson, David W. Macdonald

**Affiliations:** 1 Oxford Martin School and Department of Biology, University of Oxford, Oxford, UK; 2 Cambridge Conservation Initiative, David Attenborough Building, Cambridge, UK; 3 Wildlife Conservation Research Unit, Department of Biology, University of Oxford, Oxford, UK; 4 Department of Biological Sciences, Northern Arizona University, Flagstaff, AZ USA; 5 Eco Values for Sustainable Development, Lutfi Quder Street, 11610 Amman, Jordan; 6 Centre for Integrative Conservation, Xishuangbanna Tropical Botanical Garden, Chinese Academy of Sciences, 666303 Yunnan, China; 7 Department of Wildlife Ecology and Management, Duzce University, Duzce, 81620 Turkey; 8 Terrestrial Ecology Research Group, Technical University of Munich, 85354 Freising, Germany; 9 Wildlife Consultant, P.O Box 82, Sadah, 100 Oman; 10 Wildlife Conservation Society, Mongolia Program, Ulaanbaatar, Mongolia; 11 Institute of Zoology, Academy of Sciences of the Republic of Uzbekistan, Tashkent, Uzbekistan; 12 Directorate General of Natural Resources and Ecosystem Conservation, Indonesian Ministry of Environment and Forestry, Jakarta, Indonesia; 13 Field Research Department, Prince Saud al Faisal Wildlife Research Centre, Taif, Saudi Arabia; 14 Department of Forestry & Wildlife Management, University of Haripur, Haripur, Pakistan; 15 Department of National Parks and Wildlife Conservation, Ministry of Forests and Environment, Government of Nepal, Singhadurbar, Kathmandu Nepal; 16 Caucasus Nature Fund, Tbilisi, Georgia; 17 Vasundhara Sector 5, Ghaziabad, 201012 Uttar Pradesh India; 18 Independent Wildlife Researcher, Sulaimani, Kurdistan Region Iraq; 19 A.N. Severtsov Institute of Ecology and Evolution of the Russian Academy of Sciences, Leninsky Prospekt, 33, Moscow, 119071 Russian Federation; 20 Institute of Tropical Biodiversity and Sustainable Development, University Malaysia Terengganu, 21030 Kuala Nerus, Terengganu Malaysia

**Keywords:** Conservation biology, Biodiversity

## Abstract

Aichi Target 11 committed governments to protect ≥17% of their terrestrial environments by 2020, yet it was rarely achieved, raising questions about the post-2020 Global Biodiversity Framework goal to protect 30% by 2030. Asia is a challenging continent for such targets, combining high biodiversity with dense human populations. Here, we evaluated achievements in Asia against Aichi Target 11. We found that Asia was the most underperforming continent globally, with just 13.2% of terrestrial protected area (PA) coverage, averaging 14.1 ± SE 1.8% per country in 2020. 73.1% of terrestrial ecoregions had <17% representation and only 7% of PAs even had an assessment of management effectiveness. We found that a higher agricultural land in 2015 was associated with lower PA coverage today. Asian countries also showed a remarkably slow average annual pace of 0.4 ± SE 0.1% increase of PA extent. These combined lines of evidence suggest that the ambitious 2030 targets are unlikely to be achieved in Asia unless the PA coverage to increase 2.4-5.9 times faster. We provided three recommendations to support Asian countries to meet their post-2020 biodiversity targets: complete reporting and the wider adoption “other effective area-based conservation measures”; restoring disturbed landscapes; and bolstering transboundary PAs.

## Abstract (web summary)

Asia is shown to be behind in meeting the Aichi Target 11 under the Convention on Biological Diversity and current trends indicate that 2030 targets are unlikely to be achieved with severe impact on biodiversity.

## Introduction

Protected areas (PAs) and conserved areas are perhaps the most effective tool for safeguarding species and ecosystems globally^1^ and are therefore incorporated into multiple political targets for international conservation. Aichi Target 11 committed governments to protect at least 17% of their terrestrial area by 2020, under the Convention on Biological Diversity (CBD). The next generation of the CBD’s global goals, in draft form as the post-2020 Global Biodiversity Framework (5), includes Target 3 to protect at least 30% of the planet by 2030, with the focus on areas particularly important for biodiversity. However, repeated failures to meet previous biodiversity targets^2,3^ suggest that post-2020 agreements and targets need to be informed by past performance, not least because this indicates likelihood of future success. In particular, there is a need to identify the heterogeneous contribution made by different countries and regions to the global outcomes and where possible, predict that heterogeneity to inform future strategy.

However, a further difficulty with comparative assessment of regional and national performances against area-based targets, is that cultural and other differences can affect the reporting itself. PA coverage statistics rely on countries reporting to the World Database on Protected Areas (WDPA) and what is reported (and not reported) can vary non-randomly across regions and countries, for many reasons^4^. For example, if a country’s conservation areas include a large number of land use types or governance arrangements that do not naturally get collated by Environment Ministries attempting to report to databases mostly managed in Europe and North America. Consequently, many locally-important conservation areas may be omitted, for reasons ranging from differences in what countries choose to report as “protected areas,” to differences in the level of data that countries are able to gather about each use or governance type. Despite this cultural variation, global studies often compare regional and national performance on a target by taking all the data at face value, leading to some assessments of Global South outcomes that risk seeming post-colonial in failing to understand how Global South patterns of areal conservation can differ from those in the Global North, and then drawing negative conclusions about what has been achieved.

Asia as a continent has very high conservation value, ranking as one the richest places on earth for the diversity of living forms. It contains seven biodiversity hotspots, high levels of endemism and some of the most charismatic fauna, all living alongside over half of the world’s human population^5^. And yet, global official statistics in 2019 suggested that Asia was also one of the most underperforming continents in relation to the Aichi Target 11, with PA coverage below the global mean^5^, relatively poor representation of ecoregions inside the protected area networks, very few assessments of protected area effectiveness^1^, and low PA connectivity (3.2%) in comparison with the global mean (9.7%)^6^. Importantly, over 60% of the global population currently lives in Asia^7^, with faster than global average human population growth^8^ and consequently, increases in food production and conversion to agricultural land^9^, all leading to high rates of land clearing in PAs^10^. By 2050, Asia is projected to experience the highest rates of habitat loss from conversion to agricultural lands comparing to other continents^11^ and a further increase in its large population^8^, implying even higher human pressure. China’s Belt and Road Initiative, while potentially boosting the economy for many Asian countries, is likely to increase pressure on biodiversity and fragment PAs even more^12,13^.

Here, we first re-assess and statistically model the achievements of Asian countries against Aichi Target 11, based on those countries’ own, context-specific national understanding of what represents conservation-focused land use types (protected areas). The headline component of Aichi 11 is the area covered by PAs, but it also includes two sub-targets of representativity and PA management effectiveness (PAME), so we assess and model all three of those sub-targets. Additionally, we assess and model how much of the ranges of highly at-risk mammal species (CR/EN) are covered by Asian PAs. Finally, we quantify the rate of growth of PA area up to 2020. We then estimate the likelihood that Asia will achieve a 2030 goal for 30% PA coverage, based on a combination of all these results and models, with further examination of sub-regional patterns and likely target outcomes. Finally, we suggest a number of foci and interventions, specific to an Asia context, that could improve the future chances of success, so that this continent’s rich biodiversity can be conserved despite the strong socioeconomic challenges involved.

## Results

### Area-based sub-target

We found that 13.2% of Asian terrestrial landscapes were covered by PAs by the target date for Aichi 11 based on our in-country sources. However, it was 17.4% lower based on WDPA data (10.9%). The average increase in coverage across Asia during the 2010s was 0.4% ± SE 0.1% per year. PA coverage at the level of individual countries increased from a mean 11.1% in 2010 (SE = 1.4%) to 14.1% by 2020 (SE = 1.8%) based on our in-country sources, which was 16.5% higher than WDPA data (12.1 ± SE 1.6%). However, these overall figures concealed considerable country-level and sub-regional heterogeneity.

A total of 8,673,433 km^2^ across 10 countries, equaling 19.6% of Asian terrestrial landscapes was managed as hunting concessions, governed by governments, communities or private sectors, but these areas have not been included in the countries’ report to the Protected Planet Initiative databases. Most of these areas are locally important in terms of biodiversity conservation and local socioeconomic outcomes which may qualify them as examples of “other effective area-based conservation measures” (OECMs). The increase in area-based conservation coverage represented by these areas, above the current Protected Planet Initiative statistic, ranged from 0.2% (Iran) to 41.4% (Russia). With that update incorporated, a total of 32.9% of Asian terrestrial landscapes are under protection, either as protected areas or hunting concessions (potentially as one type of OECMs).

**Fig. 1:**
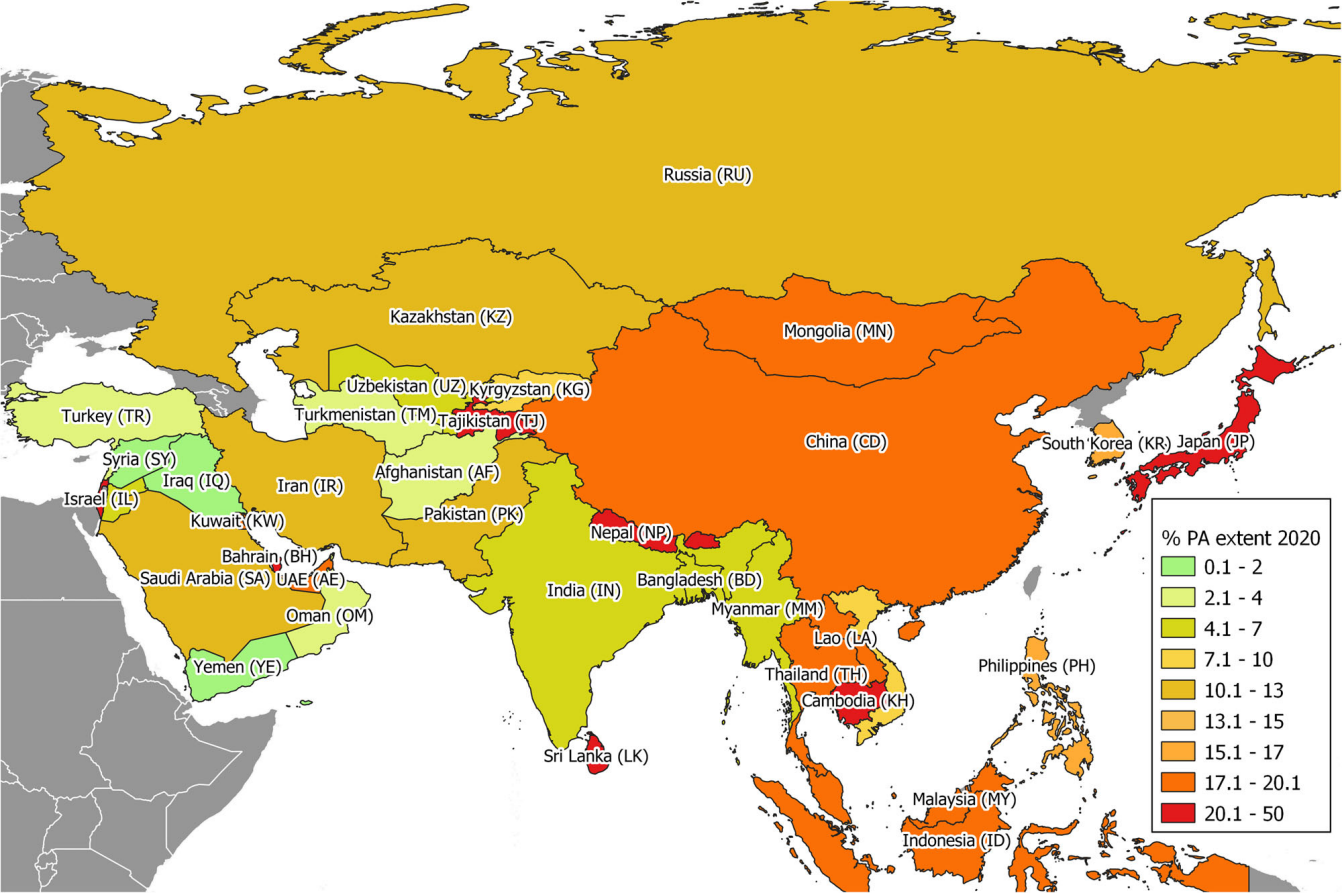
Asian countries and the percentage of protected areas (PA) in 2020. The full and abbreviated names for all Asian countries analyzed in this study are shown. The map projected using WGS 84 EPSG: 4326 coordinate reference system.

**Fig. 2:**
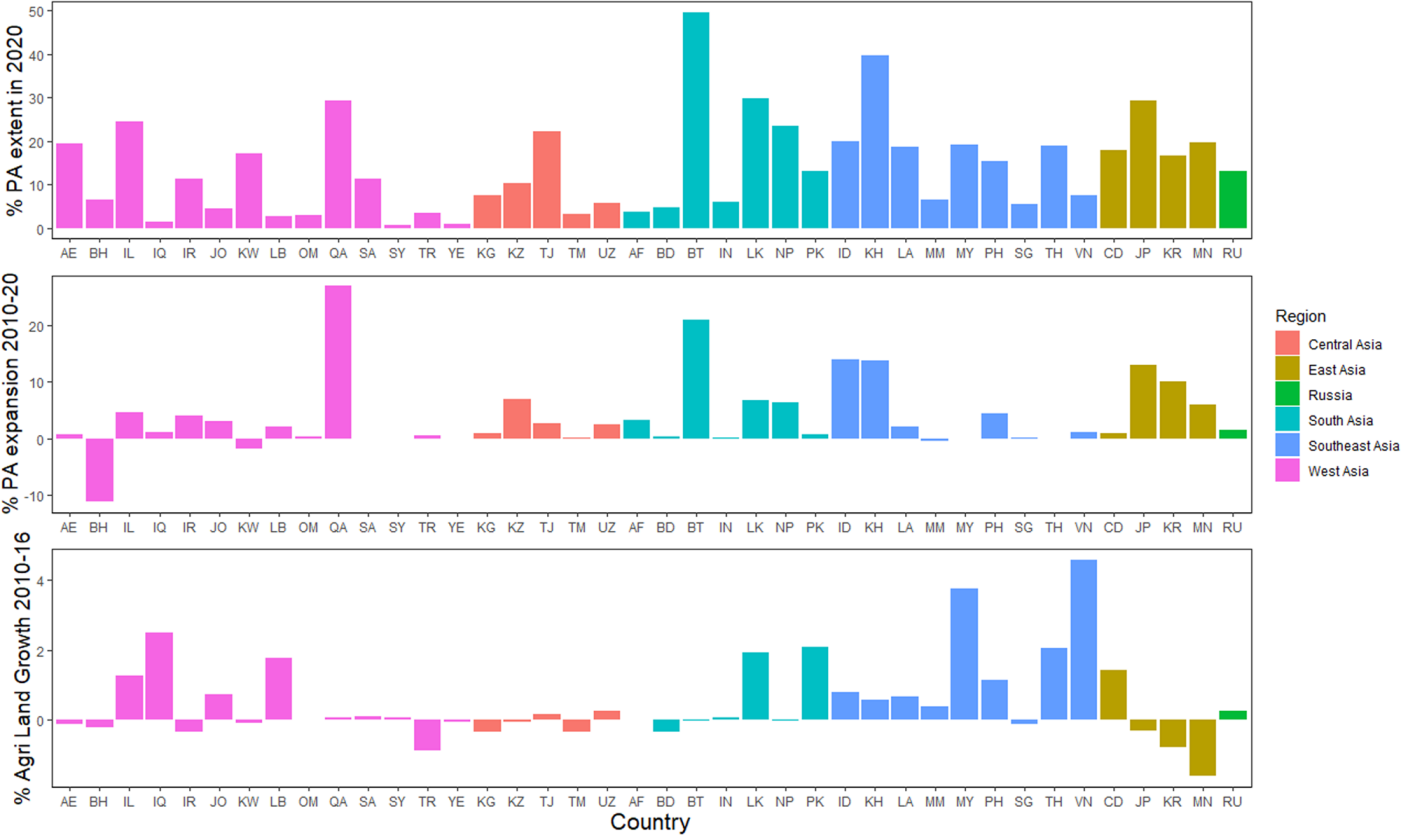
Barplots showing the arial-based metrics for 40 Asian countries analyzed in this study. Top: the percentage of PA extent in 2020; middle: the percentage of PA expansion between 2010 and 2020 and bottom: the percentage of agricultural land growth between 2010 and 2016.

We found that 40% of Asian countries met a target of 17% coverage for PAs by 2020 based on our in-country sources, mainly in East and some South Asia, whereas West and Central Asian countries had generally not achieved this target (Figs. 1 and 2). We did not find any statistically significant association between the proportions of highly at-risk (CR/EN) mammalian species range outside PAs and the % PA extent in 2020 (*β* = −0.22 ± SE 0.15, *t* = −1.51, *P* = 0.14 in a Generalized Linear Model). The highest proportions of the highly at-risk (CR/EN) mammalian species range outside PAs were seen in West (*β*_CR/EN_outsidePA_ = 1.77 ± SE 0.46, *t* = 3.86, *P* < 0.001) and East Asia (*β*_CR/EN_outsidePA_ = 2.07 ± SE 0.61, *t* = 3.39, *P* < 0.001), respectively. Indeed, the lower-diversity sub-region of East Asia showed the greatest level of protection, followed by higher-diversity Southeast Asia.

When we considered the pattern of terrestrial PA expansion for 2010-2020 (rather than the final coverage achieved), two countries in Southeast Asia (Myanmar and Thailand) showed small decreases in PA coverage (PA downgrading, degazettement and downsizing) whereas Indonesia and Cambodia showed significant (>10%) expansion. In South and West Asia, most countries showed an increase in area, with Bhutan and Qatar gaining >10%, but Kuwait lost area. In East Asia, all countries showed at least some PA expansion (South Korea and Japan by >10%) whereas in Central Asia, almost no change was seen. It is also noteworthy that between 2010 and 2015, agricultural lands increased by 2.0% across the continent, averaging 0.51 ± SE 0.03% per year at country level, although 18 counties (45.0%) had agricultural land loss, mainly in West and Central Asia (12 out of 18 countries with agricultural land loss; Fig. 2).

**Fig. 3:**
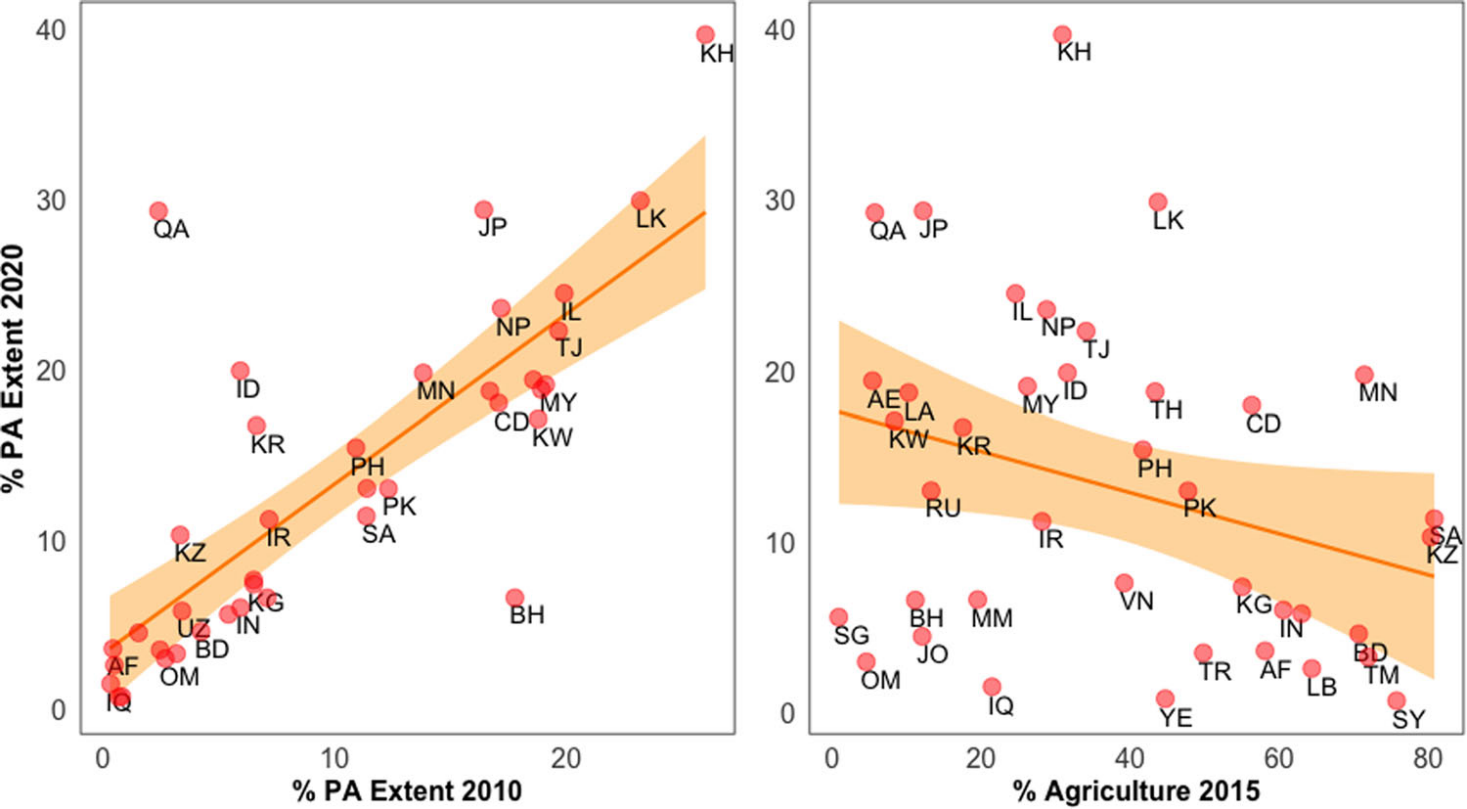
Relationship between log % of PA change during the 2010s and left) % PA extent in 2010, right) % agricultural lands in 2015 for Asian continent, based on generalized linear models. See Supplementary Table 1 for two-letter country abbreviations.

In our attempt to model the variation in achievement of area-based target (% PA extent), we found a single model with a ΔAICc weight of 1.0 (*R*^2^_adj_ = 0.66; Table 1). There was no evidence to reject the null hypothesis that the model fits well (*P* = 0.99). This model included the predictors % agricultural extent in 2015, % PA extent in 2010, and sub-region (Table 1). Specifically, the coefficients suggested that countries with greater PA extent in 2010 and a smaller percentage of agricultural lands in 2015 were more likely to achieve higher percentage of PA extent by 2020 (*β*_PAExtent2020_ = 0.58 ± SE 0.10, *t* = 5.74, *P* < 0.001 and *β*_Agriculture2015_ = −0.36 ± 0.11, *t* = −3.25, *P* = 0.003; Fig. 3). In the sub-regional effect, the smallest and largest % PA extents in 2020 were found in West (*P* = 0.01) and Southeast Asia (*P* = 0.08) respectively (Fig. 2).

**Table 1:** Results of generalized linear models testing different hypotheses on the association between the percentage of PA extent in 2020 and ecological and geopolitical factors in Asian countries.

Model	df	AICc	Delta AICc	AICc weight
%PAExtent2020 ~ Region + PAExtent2010 + Agriculture2015	10	85.9	0.0	1.0
% PAExtent2020 ~ Region + PAExtent2010 + NGO + Publications + Urgency	11	99.9	13.9	0.0
% PAExtent2020 ~ Region ++ Violence + Governance	9	106.7	20.8	0.0
% PAExtent2020 ~ Region + GDP + GDPGrowth	9	112.0	26.1	0.0
% PAExtent2020 ~ Region + Urgency	8	112.6	26.6	0.0
% PAExtent2020 ~ Region + GDP + GDPGrowth + GDP::GDPGrowth	10	113.3	27.4	0.0
% PAExtent2020 ~ Region + AgriculturalGrowth + Agriculture	9	117.2	31.2	0.0
% PAExtent2020 ~ Region	7	117.2	31.3	0.0
% PAExtent2020 ~ Region + AgriculturalGrowth + Agriculture + GDP + GDPGrowth	11	118.1	32.2	0.0
% PAExtent2020 ~ Region + NGO + Publications	9	118.9	33.0	0.0
% PAExtent2020 ~ Region + AgriculturalGrowth + Agriculture + AgriculturalGrowth::Agriculture2015	10	120.7	34.8	0.0
% PAExtent2020 ~ Region + AgriculturalGrowth + Agriculture + PAExtent2010 + GDP + GDPGrowth + NGO + Connectivity + Publications + Violence + Governance + Urgency + AgriculturalGrowth::Agriculture + GDP::GDPGrowth	19	122.1	36.1	0.0
% PAExtent2020 ~ Region + AgriculturalGrowth + Agriculture + GDP2019 + GDPGrowth + AgriculturalGrowth:: Agriculture + GDP::GDPGrowth	13	124.7	38.8	0.0

For PA growth rates, we found that with the current annual growth rate (*r*) of 0.03 for 2010–2020, Asia can achieve only 18.6% of PA extent by 2030. A 2.5 times larger *r* (0.08) would be needed to meet the 30% target by 2030 for the entire continent. The continent as a whole is therefore projected to miss a putative 30% areal target by a considerable margin. On a sub-regional level, all regions except Central Asia are projected to miss the putative 30% target, with West and South Asia as the most underperforming regions, projected to achieve 11.3 and 10.0% coverage respectively by 2030. Achieving the post-2020 Global Biodiversity Framework’s target of at least 30% of the planet protected would need 2.4 times faster PA growth for East and Southeast Asia, 4.4 times faster in West Asia and 5.9 times faster in South Asia.

### Ecological representativity sub-target

**Fig. 4:**
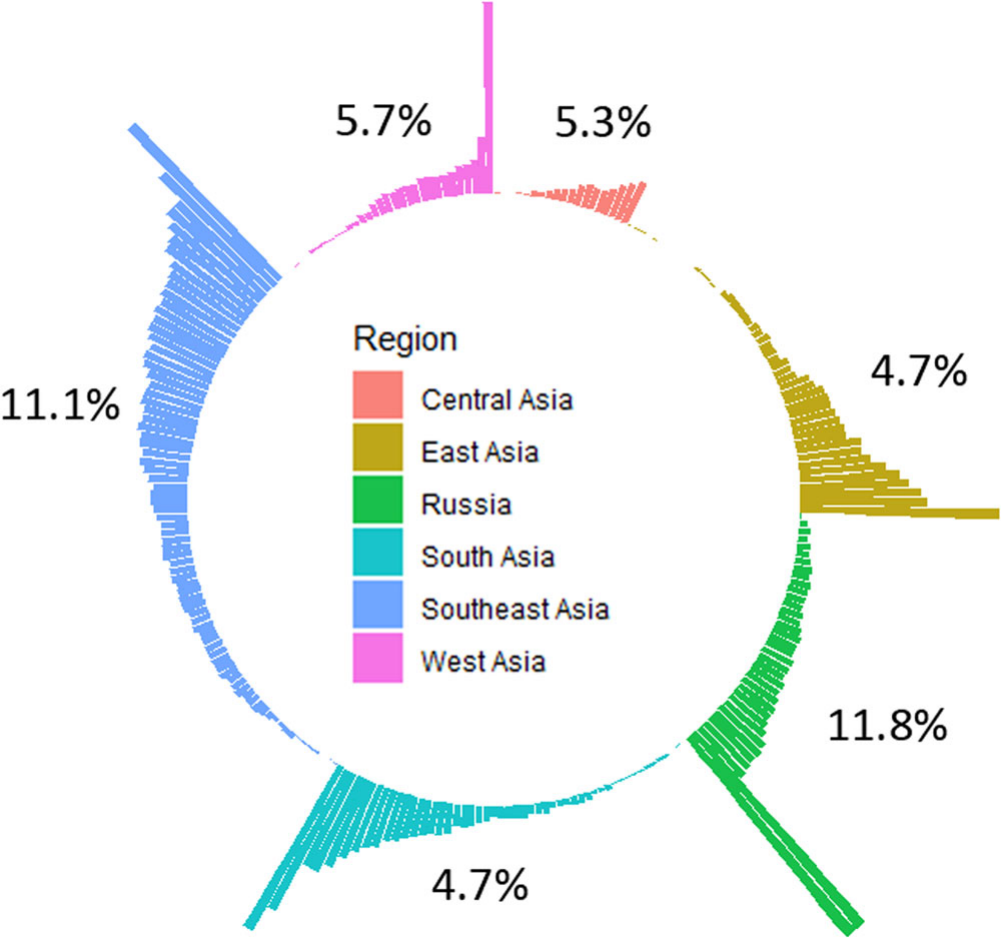
Circular barplot of the ecological representativity, defined as the percentage of ecoregions within the network of PAs for different Asian regions. Numbers denote the median of ecological representativeness per each region.

For ecological representativity, we found that 73.1% (*n* = 196) of 268 terrestrial ecoregions in Asia had less than 17% coverage inside the PA network (Fig. 4). Nonetheless, apart from Socotra Island xeric shrublands, no terrestrial ecoregion in West Asia achieved ≥17% of PA coverage. Similarly, the overlap between the PA network and the ecoregion did not exceed ≥17% in central Asia, except three ecoregions of Altai Alpine meadow and tundra, Ural montane forests and tundra and Pamir alpine desert and tundra.

In our sub-analysis of how well PA systems covered threatened species, we found that for 241 highly at-risk (CR/EN) mammalian species across Asia, a mean of 84.4% of their ranges fell outside the PA network (SE = 2.3%). A single model was selected for ecological representativeness with a weight of 0.71, without any evidence to reject the null hypothesis that the model fits well (*P* = 0.96). The model included only Region as factor (*R*^2^_adj_ = 0. 11; Table 2) but did not show any evidence for geographic differences across Asian regions (*P* > 0.05).

**Table 2:** Results of generalized linear models testing different hypotheses on the association between the percentage of ecoregions protected by the PA network in 2020 and ecological and geopolitical factors in Asian countries.

Model	df	AICc	Delta AICc	AICc weight
%ProtectedEcoregion2020 ~ Region	7	175.18	0.00	0.71
% ProtectedEcoregion2020 ~ Region + GDP + GDPGrowth	9	178.42	3.24	0.14
% ProtectedEcoregion2020 ~ Region + AgriculturalGrowth + Agriculture	9	180.03	4.85	0.06
% ProtectedEcoregion2020 ~ Region + NGO + Publication	9	181.12	5.95	0.04
% ProtectedEcoregion2020 ~ Region + GDP + GDPGrowth + GDP::GDPGrowth	10	182.07	6.89	0.02
% ProtectedEcoregion2020 ~ Region + AgriculturalGrowth + Agriculture + AgriculturalGrowth::Agriculture	10	183.34	8.16	0.01
% ProtectedEcoregion2020 ~ Region + GDP + GDPGrowth + AgriculturalGrowth + Agriculture	11	183.83	8.65	0.01
% ProtectedEcoregion2020 ~ Region + NGO + Publication + %PAExtent2020	10	184.59	9.42	0.01
% ProtectedEcoregion2020 ~ Region + AgriculturalGrowth + Agriculture + GDP + GDPGrowth + NGO + Publication	13	191.82	16.65	0.00
% ProtectedEcoregion2020 ~ Region + AgriculturalGrowth + Agriculture + GDP + GDPGrowth + NGO + Publication + GDP::GDPGrowth + AgriculturalGrowth::Agriculture + %PAExtent2020	16	206.20	31.03	0.00

For the coverage of highly at-risk (CR/EN) mammalian species, a single statistical model was also selected, with non-significant deviance goodness of fit (*P* = 0.83), which included only the % PA extent by 2020 and Region as predictors (*R*^2^_adj_ = 0. 27). Although there was no evidence for association between the % PA extent by 2020 and the coverage of threatened species (*β*_PAExtent2020_ = −0.23 ± SE 0.15, *t* = −1.57, *P* = 0.13). However, the coverage of threatened species varied geographically, with high intercept differences for East Asia (*β*_EastAsia_ = −0.23 ± SE 0.15, *t* = −1.57, *P* = 0.13), implying the largest median of range of highly at-risk (CR/EN) mammalian species outside the current network of PAs within each country.

### PA management effectiveness sub-target

For the level of PAME assessment, we found that out of 22781 PAs within the 40 studied Asian countries, only 7.0% have been assessed based on PAME criteria (n = 1599), averaging 17.4% ± of PAs per country (SE = 2.5%). Israel, Japan, Lao, Bahrain, Oman and Qatar had no PA assessed based on the PAME criteria while over 1/3 of PAs in Indonesia, Cambodia, Bhutan, Jordan, Nepal, Turkey, Singapore and the UAE were PAME assessed. When modeling the level of PAME assessment, three best supported models were averaged (Table 3), with the averaged model including GDP2019, % PA extent 2020 and the Region as predictors. The averaged model coefficients would be non-significant under a hypothesis-testing approach (*β*_GDP2019_ = −0.18 ± SE 0.12, *t* = 1.47, *P* = 0.14 and *β*_PAExtent2020_ = −0.15 ± SE 0.11, *t* = 1.31, *P* = 0.19). Similarly, there was no evidence for the association between the ratio of PAs with PAME and Asian regions (*P* > 0.05).

**Table 3:** Results of generalized linear models testing different hypotheses on the association between the ratio of PAs with management effectiveness (PAME) in 2020 and ecological and geopolitical factors in Asian countries.

Model	df	AICc	Delta AICc	AICc weight
PAME-ratio2020 ~ Region + GDP	8	63.17	0.00	0.34
PAME-ratio2020 ~ Region	7	63.80	0.63	0.25
PAME-ratio2020 ~ Region + %PAExtent2020	8	65.00	1.83	0.14
PAME-ratio2020 ~ Region + GDPGrowth	8	65.76	2.58	0.09
PAME-ratio2020 ~ Region + GDP + GDPGrowth	9	66.55	3.38	0.06
PAME-ratio2020 ~ Region + NGO	8	67.24	4.06	0.05
PAME-ratio2020 ~ Region + Publication	8	67.30	4.13	0.04
PAME-ratio2020 ~ Region + Publication + NGO	10	70.37	7.20	0.01
PAME-ratio2020 ~ Region + NGO + Publication + %PAExtent2020	9	71.06	7.89	0.01
PAME-ratio2020 ~ Region + GDP + GDPGrowth + GDP::GDPGrowth + NGO + Publication + %PAExtent2020	10	71.78	8.60	0.00

## Discussion

We showed that Aichi target 11 has failed to garner traction across most of Asia, with many countries showing little or no expansion in their PAs, the exception being countries in East Asia. Only 40% of Asian countries met their target of 17% coverage for PAs by 2020. On average, Asian countries achieved 14.1% PA coverage by 2020 (± SE 1.8%), 1.1 percentage points less than the current global mean of 15.2%^5^ and 2.9 percentage points slower than the minimum required under Aichi Target 11 (17%), with a remarkably slow average annual growth of 0.4 ± SE 0.1% in PA extent. For the representativity sub-target, only 26.9% of Asian ecoregions reached ≥17% protection by 2020, compared to the global average of 42.6%^14^. For the ‘effective management’ sub-target, Asia was also the most underperforming continent in the world, with the lowest percentage of PAs for which assessments of effectiveness (only 7.0%)^5^ have been reported, compared to a global mean of 9.1%^15^. These levels of low comparative performance held true despite our major expansion of the officially reported data on PA coverage which included new areas, such as community and government hunting areas, that were of considerable cultural and biodiversity relevance in areal conservation but were not listed in the official Protected Planet Initiative databases (i.e., the database used to assess progress against international area-based conservation targets).

If we use the patterns observed to assess likely performance against a proposed 30% area-based target by 2030 (Target 2), the clear implication is that Asia is likely to fall even further short (with similarly poor outcomes expected for representativity and, if quantified as a target, for management effectiveness). The evidence from our statistical models suggest that past coverage is a strong predictor of future coverage, and 2020 areal achievements were generally below the target of 17%. The percentage of national territory dedicated to agriculture also predicted PA coverage, and post-2020, Asia is projected to experience the highest rates of habitat loss from conversion to agricultural lands^11^. Indeed, given the rapid expected increase in human pressure and associated land conversion, it seems likely that areal coverage could lag targets by even more in 2030 than it did in 2020. These conclusions are supported by our PA-growth analysis, where we found that to achieve 2030 values of 30%, PAs would need to expand at least twice as fast and in some regions, nearly six times as fast as they achieved during the previous (2010–2020) target period.

Our updates to official PA coverage statistics confirm that PA coverage is underreported by 16.5% on country-level. The most obvious way of improving coverage statistics is therefore simply more complete reporting. For example, some countries may share only a subset of their PA types or layers with the WDPA. Importantly, a drive for better reporting could include greater political inclusivity and recognition of the contribution of non-state actors to areal protection. In particular, the PAs included in national reporting to WDPA are often those owned or managed by government. However, it is likely there are many areas outside of the PA network that are being conserved by a diverse set of actors, including private and community PAs, which have not yet been recognized and reported. Many such areas could be regarded as examples of “other effective area-based conservation measures” (OECMs), which have always been part of Aichi Target 11^16^. Indeed, some of the countries that perform worst under traditional measures of PA coverage may in fact have some of the larger areas of non-traditional coverage. For example, our data showed that the inclusion of hunting concessions (Supplementary Table 1), which were detected mostly in West and Central Asian countries that did not achieve the 17% areal target for Aichi 11, bolstered those countries’ area-based achievements moderately. Although some may argue that hunting concessions can contribute to the rural socio-ecological resilience for remote mountain communities and offer economic incentives for an integrated conservation and development paradigm to combat illegal wildlife trade^17,18^. It is also clear, however, that hunting concessions can be compromised by ethical, ecological and socioeconomic concerns^19,20^. It would be prudent to precede any expansion of hunting concessions with a rigorous evaluation of these concerns as they apply to any area proposed for expansion, with a focus on the likelihood of adverse effects for wildlife and human livelihoods.

Such areas can be added to coverage statistics by reporting them to the Protected Planet Initiative’s World Database on Other Effective Area-Based Conservation Measures (WD-OECM). The formal current definition of OECMs is “geographically defined areas other than PAs, governed to achieve positive biodiversity conservation outcomes with associated ecosystem functions and services as well as cultural, spiritual, socio–economic, and other locally relevant values”^21^. OECMs may be managed for many different objectives but they must deliver effective conservation^5^ and can therefore provide the opportunity for formal support for areas delivering conservation outcomes outside the PA network^22^. A key requirement is a need for review and recognition of OECMs within national policy frameworks, which will facilitate the reporting to the Protected Planet Initiative based on what is included in national accountings. Many OECMs already identified are implemented by a diverse set of actors, including indigenous peoples, local communities, and the private sector. They therefore offer the opportunity to engage and support rights-holders and stakeholders beyond government agencies, and to promote more equitable partnerships in global conservation efforts, meeting other CBD objectives that relate to the area-based targets^5^.

We support ambitious planning for the post-2020 Global Biodiversity Framework^3,23,24^. Nonetheless, expanding the PA network in Asia needs to be coupled by obtaining the support of local communities through considering the well-being of people and reducing human pressures^25,26^. However, taking all these lines of evidence together, achieving a post-2020 Target 2 of 30% coverage^5^ seems very unlikely without a major change in ambition and a range of dedicated interventions, both at the levels of countries and ecoregions. In addition to improved PA reporting and the identification and inclusion of the OECMs, we offer two inter-related suggestions which deserve further exploration in how they can potentially support Asian countries in the aspiration to achieve more under their post-2020 biodiversity commitments:

1. Restoring disturbed landscapes: Large-scale farmland abandonment, as an opportunity for restoration and biodiversity regeneration^27^, has occurred in Asia^28,29^, and 45.0% of Asian countries had agricultural land loss based on our analysis. Our analysis showed that most farmland abandonment were also concentrated in the West and Central Asian regions where performance against PA targets was worst. Equally important, lowland tropical rainforest landscapes of East Asia also provide potential restoration opportunities. Although these areas have higher potential return of benefits and feasibility^30^; however, the existing efforts are hampered by local governance and power imbalance between stakeholders^31^ as well as the development of plantations of fast-growing species for timber production rather than landscape restoration^32^. Nonetheless, the restoration cannot necessarily offset loss of original ecosystems.
2. Promoting transboundary PAs: Asia contained approximately 82% of global border hotspots (the richest 5% of border segments) for threatened transboundary species^33^, with some species whose persistence depends on transboundary areas^34^. Asian countries have larger protected areas with higher potential connectivity near borders^35^. A quantified area-based baseline could be included in future targets, reflecting and motivating the potential contribution of transboundary areas towards countries’ commitments.

Area-based targets remain the cornerstone for countries’ biodiversity commitments^36^. However, it is also important to include “performance based” sub-targets in any future Target 3, emphasizing the need not simply to increase area, but also to make sure that any expanded areas are performing effectively against an overarching goal of protecting biodiversity. Effectively structured and clearly framed targets allow the translation of targets into actionable policies^37^. For example, the clearly defined area-based element of Target 11 had high compliance with criteria such as measurability, unambiguity, realism, and scalability, whereas 4 qualitative elements were substantially compliant^37^. Proposed additional performance-based sub-targets include “biodiversity value”^3^ and a requirement that 70% of ecoregions should meet a 90% target for the Biodiversity Intactness Index (implying that abundances across all functional groups would recover to near-preindustrial levels by 2050^23,38^). Some scholars have also proposed intactness metrics^39^, for example it was highlighted the region still hosts high diversity despite having fragmented land cover^40,41^. It is therefore important not only to consider apparent “intactness” but also to use more suitable measures of biodiversity. Intactness particularly has value as a metric when determining whether restoration or preservation is more appropriate, and could be retained for this and other purposes.

Monitoring is also needed to track interim progress and targets and sub-targets (not least because we manage what we measure), and could itself be a sub-target. However, the capacity needed to monitor and evaluate performance-based targets is unlikely to exist in many Asian countries, on the evidence that they have the fewest PAs evaluated based on PAME criteria compared to other continents, and that the majority of Asian countries appear to be relatively understudied with respect to biodiversity^42^. Ways to improve this could include international collaboration with Asian countries, increasing partnerships, and capacity building for Asian scholars and conservationists to bolster biodiversity research and monitoring. Also, creating a digital infrastructure to operationalize national-level data capture for monitoring of both protected area management and trends in species and ecosystems based on field observations and remote sensing can promote effectiveness^43^.

Asia is a complex region, as it is the only region where European languages are rarely fluent. The tendency for conventions to limit languages available is a barrier to engagement in these regions, and that UN conventions should use all UN languages as a standard minimum for biodiversity related conventions to encourage engagement from this diverse, yet neglected region of the planet. Some countries also have weaker governance institutions and unreliable monitoring and evaluation measures to track their impact or success at the local level. Therefore, it is unlikely that a “one size fits all” approach can be successfully implemented in different regions. With great variability in human pressure, biodiversity richness and geopolitical realms across Asia, careful planning is therefore needed to meet post-2020 area-based biodiversity targets (≥30% by 2030) in balance with human demands.

## Methods

We first quantified the areal coverage achieved by 2020 and compared it to the 17% commitment to in Aichi Target 11. Although Aichi Target 11 might protect more biodiversity if targets were different in each country, it was widely interpreted as a national target (as indeed is occurring with the post-2020 Target 2), and so we follow that practice and ask whether countries reached 17% coverage individually. We started by extracting the percentage of national terrestrial land occupied by PAs as of end 2020 from the World Database on Protected Areas (WDPA)^44^. However, to develop a more Asian perspective, and also to compensate for time lags and omissions in reporting to the WDPA^4^, we checked the latest available data from WDPA^5^ against several in-country sources concerning PA extent in 2020, updating where necessary (Supplementary Table 2). In particular, we obtained the area of community or private controlled hunting concessions within Asian countries with active trophy hunting programs from national sources as a measure of potential additional contribution to countries area-based targets. We excluded any countries for which complete, robust data were lacking. In total, 40 Asian countries were retained in the final analyses (Supplementary Table 1). Although Russia and Kazakhstan span Eurasia, we included it as an Asian country because all predictor covariates were available only at country scale, not for a subset of the country.

For the ecological representativity sub-target, we used coverage of terrestrial ecoregions, defined as large areas with distinct biodiversity values which are widely used for tracking progress towards ecological representativeness of Aichi Target 11^14,45^. We used the PA network in each Asian country to calculate the percentage of each ecoregion outside that network, and then took the median of these values across all the ecoregions found in each country as our representativeness metric. These calculations were conducted using Spatial Analyst toolbox in ArcGIS 10.3^46^ based on shapefile of terrestrial ecoregions obtained from The Nature Conservancy Geospatial Conservation Atlas (geospatial.tnc.org). For coverage of highly at-risk mammalian species, we took species ranges from IUCN spatial layers (from www.redlist.org) and calculated the median percentage of all Endangered (EN) and Critically Endangered (CR) species that was overlapped by our updated PA spatial layer.

For the effective management sub-target of Aichi Target 11, we used Protected Area Management Effectiveness (PAME) assessments (22), which indicate how well a PA is managing conservation values (e.g. biodiversity conservation, ecosystem service and cultural service provision) and achieving stated goals and objectives^47^. However, there were very few PAME assessments in many countries, making the PAME scores themselves patchy and poorly informative. To indicate progress on effectiveness, we therefore focused on quantifying the percentage of PAs that had carried out (and reported) an assessment in each country. We obtained PAME scores from the Global Database on Protected Area Management Effectiveness (PAME)^5^ and calculated the mean of each country’s PAs’ scores evaluated based on PAME methodologies. For Turkey, there was a substantial mismatch between the number of PAs reported on WDPA database (*n* = 18) and the Turkish Ministry of Forestry (*n* = 929)^48^. To keep consistency across the Asian countries, we used the WDMA data to calculate the ratio of PAME assessments.

We then developed predictive models that aimed to explain the variation in performance against these metrics. We focused on modeling the areal coverage target (13 candidate models) because this was our main metric of interest in this study, and because this was the metric for which there was a full set of data on both current patterns and detailed historical change. Using Generalized Linear Models (GLMs), we tested nine national-level predictors (Supplementary Table 2), broadly categorized as ecological, relating to conservation capacity, or geopolitical status. Specifically, we theorized that the PA and coverage outcomes might be positively associated with a nation’s wealth and development (GDP)^49^, good management^36^ and the quality of governance (measured as the average of the six Worldwide Governance Indicators)^50^, technical capacity (measured by NGO support levels and conservation publications)^42^ and conservation urgency (Supplementary Table 2), and negatively associated with human demands on land use (% agricultural land and rate of growth in agricultural land)^51^ and the frequency of armed conflicts^52^. In addition, we hypothesized that the area already protected at the beginning of the Aichi target period (2010–2020) might influence the final performance against Target 11, and added coverage in 2010 as a term in our models. However, following the ratification of the Aichi Targets in 2010, it took a few years for countries to update their PA status on WDPA database, resulting in 2014 as the earliest year for comprehensive data available for all Asian countries. We therefore took the 2014 values as our 2010 extent baseline. We then assumed that for all other predictor variables, 2020 outcomes may have been driven by conservation actions occurring 5–10 years earlier, following (21), and used variable values for the 2010–2015 period (i.e., averages for years as close as possible to that period, depending on data availability, Supplementary Tables 1 and 2).

We also created models to test whether variation in ecosystem representation, or in the level of PAME assessments, was related to regional differences or to socioeconomic factors. These models were based on the same predictor set as PA coverage excepting that they lacked a term for earlier values of the metric (e.g., levels of PAME assessments in 2010), not least because early values would be difficult to calculate and interpret robustly.

All statistical analyses were run in R 1.1.456^53^. All predictor variables were *z*-standardized to put effect sizes on a common scale and to give a meaningful interpretation for intercept value. We addressed collinearity between covariates using the variance inflation factor (VIF). We sequentially removed the collinearity by deleting each variable for which the VIF value was highest until all remaining VIFs were below 3^54^. We carried out a log transformation on the response variable. We also used “MuMIn” package for multi-model inference with AICc for ranking models^55^. We used model averaging to produce a single set of coefficient estimates using all models with ΔAICc < 2 from the model with the lowest AICc. Next, we explored the goodness of the fit of the model via the deviance goodness of fit test.

The ability to grow PA coverage to 30% also depends on the rate of change in coverage achievable, so we further analyzed historical rates of PA growth and applied them to project future coverage levels. We therefore calculated the exponential growth rate (*r*) implied by change of extent between 2010 and 2020, for Asia as a whole and also for as its five main regions and applied the same rate outwards to 2030. We then compared this with the rate that would require to achieve 30% by 2030. We did not attempt to project future values for management effectiveness or ecosystem representation targets because the nature of those future sub-targets remains unclear, and because achievement would depend on a number of factors not amenable to our statistical analysis.

### Reporting summary

Further information on research design is available in the Nature Research Reporting Summary linked to this article.

## Supplementary information

Supplementary Information

Reporting summary

## Data Availability

All the data used are given in Supplementary Information. All the geospatial data associated with the results presented are available on https://figshare.com/s/9615e7f167c0b4ffa572.
