# Supplementary material for: Current trends suggest most Asian countries are unlikely to meet future biodiversity targets on protected areas
Source: Commun Biol. 2022 Nov 29;5:1221. doi: 10.1038/s42003-022-04061-w (PMC9705440; doi:10.1038/s42003-022-04061-w)
Supplement: Supplementary file 1 — Supplementary Information [file 42003_2022_4061_MOESM1_ESM.pdf]

Supplementary Information

Current trends suggest most Asian countries are unlikely to meet future biodiversity targets on protected areas

Mohammad S. Farhadinia[1 *], Anthony Waldron[2], Żaneta Kaszta[3, 4], Ehab Eid[5], Alice Hughes[6], Hüseyin Ambarlı[7, 8], Hadi Al- Hikmani[9], Bayarbaatar Buuveibaatar[10], Mariya A. Gritsina[11], Iding Haidir[3, 12], Zafar-ul Islam[13], Muhammad Kabir[14], Gopal Khanal[15], Maxim A. Koshkin[16], Rahim Kulenbekov[16], Zairbek Kubanychbekov[16], Aishwarya Maheshwari[17], Ugyen Penjor[3], Hana Raza[18], Tatjana Rosen[16], Anna Yachmennikova[19], Viatcheslav V. Rozhnov[19], Nobuyuki Yamaguchi[20], Paul J. Johnson[3], David W. Macdonald[3]

[1] Oxford Martin School and Department of Biology, University of Oxford, Oxford, UK

[2] Cambridge Conservation Initiative, David Attenborough Building, Cambridge, UK

[3] Wildlife Conservation Research Unit, Department of Biology, University of Oxford, Oxford, UK

[4] Department of Biological Sciences, Northern Arizona University, Flagstaff, Arizona, USA

[5] Eco Values for Sustainable Development. Lutfi Quder Street 11610, Amman, Jordan

[6] Centre for Integrative Conservation, Xishuangbanna Tropical Botanical Garden, Chinese Academy of Sciences, Yunnan, 666303, China

[7] Department of Wildlife Ecology and Management, Duzce University, Duzce, 81620, Turkey

[8] Terrestrial Ecology Research Group, Technical University of Munich, 85354 Freising, Germany

[9] Wildlife consultant, P.O Box 82 Sadah 100 Oman

[10] Wildlife Conservation Society, Mongolia Program, Ulaanbaatar, Mongolia

[11] Institute of Zoology, Academy of Sciences of the Republic of Uzbekistan, Tashkent, Uzbekistan

[12] Directorate General of Natural Resources and Ecosystem Conservation, Indonesian Ministry of Environment and Forestry, Jakarta, Indonesia

[13] Field Research Department, Prince Saud al Faisal Wildlife Research Centre, Taif, Saudi Arabia

[14] Department of Forestry & Wildlife Management, University of Haripur, Haripur, Pakistan

[15] Department of National Parks and Wildlife Conservation, Ministry of Forests and Environment, Government of Nepal, Singhadurbar Kathmandu, Nepal

[16] Caucasus Nature Fund, Tbilisi, Georgia

[17] Vasundhara sector 5, Ghaziabad - 201012, Uttar Pradesh, India

[18] Independent wildlife researcher, Sulaimani, Kurdistan Region, Iraq

[19] A.N. Severtsov Institute of Ecology and Evolution of the Russian Academy of Sciences, Russia, 119071, Moscow, Leninsky Prospekt, 33, Russian Federation

[20] Institute of Tropical Biodiversity and Sustainable Development, University Malaysia Terengganu, 21030 Kuala Nerus, Terengganu, Malaysia

* Corresponding author email: mohammad.farhadinia@biology.ox.ac.uk (ORCID ID: 0000-0002-5385-6254)

Supplementary Table 1 List of variables evaluated in this study for 40 Asian countries in regard to the Aichi Target 11

**Country**	**Abbreviation**	**Region**	**Trophy hunting area (km^2)**	**PAME ratio 2020**	**%PA extent 2010**	**%PA extent 2020**	**%PA change**	**International support**	**Violence**
Afghanistan	AF	South Asia	0	0.13	0.4	3.6	3.2	1	126575
Bahrain	BH	West Asia	0	0.00	17.8	6.6	-11.2	1	26
Bangladesh	BD	South Asia	0	0.02	4.2	4.6	0.4	2	5506
Bhutan	BT	South Asia	0	0.36	28.8	49.7	20.9	3	0
Cambodia	KH	Southeast Asia	0	0.36	26	39.7	13.7	6	11
China	CD	East Asia	0	0.16	17.1	18	0.9	4	11
India	IN	South Asia	0	0.31	5.9	6	0.1	4	7867
Indonesia	ID	Southeast Asia	0	0.35	5.9	19.9	14	6	80
Iran	IR	West Asia	3000	0.11	7.2	11.2	4	0	510
Iraq	IQ	West Asia	0	0.04	0.3	1.5	1.2	1	59859
Israel	IL	West Asia	0	0.00	19.9	24.5	4.6	1	2019
Japan	JP	East Asia	0	0.00	16.4	29.4	13	4	0
Jordan	JO	West Asia	0	0.39	1.5	4.5	3	1	35
Kazakhstan	KZ	Central Asia	1008213	0.15	3.3	10.3	7	2	0
Kuwait	KW	West Asia	0	0.03	18.8	17.1	-1.7	1	26
Kyrgyzstan	KG	Central Asia	144000	0.14	6.5	7.4	0.9	2	44
Lao	LA	Southeast Asia	0	0.00	16.7	18.7	2	3	0
Lebanon	LB	West Asia	0	0.29	0.5	2.6	2.1	1	997
Malaysia	MY	Southeast Asia	0	0.03	19.1	19.1	0	4	71
Mongolia	MN	East Asia	213,455	0.29	13.8	19.8	6	3	0
Myanmar	MM	Southeast Asia	0	0.09	7.1	6.6	-0.5	4	5919
Nepal	NP	South Asia	1,325	0.41	17.2	23.6	6.4	3	0
Oman	OM	West Asia	0	0.00	2.7	3	0.3	0	0
Pakistan	PK	South Asia	17856.6	0.01	12.3	13	0.7	3	25607
Philippines	PH	Southeast Asia	0	0.03	10.9	15.4	4.5	4	5506
Qatar	QA	West Asia	0	0.00	2.4	29.3	26.9	1	0

Russia	RU	Russia	7086737	0.00	11.4	13	1.6	1	2197
Saudi Arabia	SA	West Asia	0	0.04	11.4	11.4	-19.9	1	239
Singapore	SG	Southeast Asia	0	0.50	5.4	5.6	0.2	3	0
South Korea	KR	East Asia	0	0.21	6.6	16.7	10.1	2	4
Sri Lanka	LK	South Asia	0	0.00	23.2	29.9	6.7	2	8
Syria	SY	West Asia	0	0.11	0.7	0.7	0	1	347254
Tajikistan	TJ	Central Asia	10,000	0.15	19.7	22.3	2.6	2	133
Thailand	TH	Southeast Asia	0	0.16	18.9	18.8	-0.1	4	1348
Turkey	TR	West Asia	332846	0.44	2.5	3.52	1.02	1	4900
Turkmenistan	TM	Central Asia	0	0.28	3.2	3.3	0.1	0	0
UAE	AE	West Asia	0	0.51	18.6	19.4	0.8	1	0
Uzbekistan	UZ	Central Asia	0	0.22	3.4	5.8	2.4	0	0
Viet Nam	VN	Southeast Asia	0	0.23	6.5	7.6	1.1	5	0
Yemen	YE	West Asia	0	0.40	0.8	0.8	0	0	24618

Supplementary Table 1 List of variables evaluated in this study for 40 Asian countries in regard to the Aichi Target 11 – *continued*

Country	Region	% Protected Ecoregions 2020	Publication	Conservation urgency	WGI	% Agriculture 2015	Agricultural growth	GDP 2019	GDP Growth
Afghanistan	South Asia	94.58	1289	53.19	-1.6	58.1	0.0	502.1	20.5
Bahrain	West Asia	0.001	249	100	-0.2	11.1	-0.2	23504.0	50.0
Bangladesh	South Asia	86.64	8188	96.35	-0.8	70.6	-0.4	1698.3	162.5
Bhutan	South Asia	51.19	597	56.67	0.6	13.6	0.0	3243.2	58.1
Cambodia	Southeast Asia	62.19	1810	63.82	-0.7	30.9	0.6	1510.3	141.0
China	East Asia	93.96	97892	99.82	-0.3	56.2	1.4	9770.8	135.6
India	South Asia	86.78	40480	86.07	-0.1	60.4	0.0	2010.0	71.6
Indonesia	Southeast Asia	82.35	8540	85.79	-0.2	31.5	0.8	3893.6	48.2
Iran	West Asia	88.07	13162	83.5	-0.9	28.2	-0.4	5627.7	-8.5
Iraq	West Asia	93.84	2078	91.1	-1.5	21.4	2.5	5834.2	69.0
Israel	West Asia	85.33	8784	58.01	0.7	24.6	1.3	41719.7	68.8
Japan	East Asia	70.38	38855	73.56	1.4	12.3	-0.3	4241.8	-10.8
Jordan	West Asia	94.1	2810	98.43	-0.1	12.0	0.7	39290.0	63.2
Kazakhstan	Central Asia	92.71	1474	92.69	-0.4	80.4	-0.1	9814.8	21.7
Kuwait	West Asia	55.23	1295	60.77	-0.2	8.4	-0.1	32032.0	16.8
Kyrgyzstan	Central Asia	82.03	447	0	-0.7	55.0	-0.4	1279.9	76.3
Lao	Southeast Asia	72.58	1765	70.68	-0.7	10.3	0.6	2534.9	155.0
Lebanon	West Asia	96.8	1443	99.9	-0.8	64.3	1.8	7784.3	38.8
Malaysia	Southeast Asia	90.43	7715	88.23	0.3	26.3	3.8	11373.2	43.0
Mongolia	East Asia	69.24	4477	86.65	0.0	71.5	-1.6	4121.7	92.7
Myanmar	Southeast Asia	71	1820	89.93	-0.9	19.5	0.4	1326.0	53.6
Nepal	South Asia	70.44	4867	69.06	-0.7	28.7	0.0	1033.9	91.5
Oman	West Asia	96.06	1474	93.83	0.2	4.6	0.0	16415.2	34.9
Pakistan	South Asia	79.97	8740	88.19	-1.0	47.8	2.1	1482.4	57.0
Philippines	Southeast Asia	76.06	6193	85.06	-0.3	41.7	1.1	3485.1	80.8
Qatar	West Asia	80.33	361	82.7	0.3	5.8	0.0	64781.7	46.6

Russia	Russia	82.72	10364	93.5	-0.7	13.3	0.2	11288.9	11.5
Saudi Arabia	West Asia	88.53	3722	89.48	-0.3	80.8	0.1	23339.0	50.1
Singapore	Southeast Asia	93.65	3965	99.97	1.6	0.9	-0.1	65233.3	55.1
South Korea	East Asia	99.96	10645	100	0.8	17.4	-0.8	31762.0	43.6
Sri Lanka	South Asia	77.17	2998	69.5	-0.1	43.7	1.9	4102.5	48.1
Syria	West Asia	99.98	1095	99.27	-2.0	75.8	0.1	NA	NA
Tajikistan	Central Asia	73.2	463	0	-1.2	34.1	0.2	826.6	43.9
Thailand	Southeast Asia	72.65	13098	60.22	-0.3	43.3	2.1	7273.6	59.4
Turkey	West Asia	99.62	22900	100	-0.5	49.8	-0.9	9370.2	-2.3
Turkmenistan	Central Asia	91.02	285	79.7	-1.4	72.0	-0.3	6966.6	80.5
UAE	West Asia	85.8	1229	93.28	0.6	5.5	-0.1	43005.0	45.3
Uzbekistan	Central Asia	85.69	567	100	-1.0	62.9	0.2	1532.4	24.1
Viet Nam	Southeast Asia	71.44	7740	77.34	-0.3	39.3	4.6	2566.6	125.9
Yemen	West Asia	62.43	741	100	-1.9	44.6	-0.1	968.2	-10.7

Supplementary Table 2 Descriptions and sources of the response and explanatory variables used in the analyses; IUCN =International Union for Conservation of Nature; NGO = non-governmental organization; PA = Protected Area; CBD = Convention of Biological Diversity.

**Variable category**	**Variable name**	**Description and source**
Response	% PA extent 2020	Extent (%) to which each country's land was covered by the network of PAs in 2020, obtained from the World Database on Protected Areas (WDPA) which is the most comprehensive global database on terrestrial and marine protected areas. The WDPA is managed by the United Nations Environment World Conservation Monitoring Centre [1]. Due to time lag in reporting to WDPA by countries, we updated each country's metric from national resources (see Supplementary Table 1).
Response	PA management effectiveness (PAME)	PA management effectiveness (PAME) assessments were obtained from the Global Database on Protected Area Management Effectiveness (25). It indicates how well a PA is being managed: primarily the extent to which management is protecting PA values (e.g. biodiversity conservation, ecosystem service and cultural service provision) and achieving stated goals and objectives, using methodologies ranging from questionnaires to more complex approaches (9).
Response	% Protected Ecoregion 2020	The median percentage of range of all ecoregions outside the current network of PAs within each country, representing ecological representativeness. Terrestrial ecoregions layer was obtained from https://geospatial.tnc.org/datasets/7b7fb9d945544d41b3e7a91494c42930_0
Ecological	% PA extent 2010	Extent (%) to which each country's land was covered by the network of PAs in 2014. We considered this as the baseline for Aichi Target 11, as it was the earliest available database of PA coverage for each country, obtained from the WDPA [1].
Ecological	Conservation urgency	The median percentage of range of highly conservation-dependent mammalian species, i.e. Critically Endangered (CR) or Endangered (EN) outside the current network of PAs within each country. Species range layer was obtained from www.redlist.org
Capacity	Publication	The number of papers labelled in the biodiversity and conservation category in the Web of Science catalogues Science Citation Index (SCI) and Social Science Citation Index (SSCI) for articles published from 1993 to 2016 that included country name, obtained from [2].
Capacity	International support	The cumulative number of world's big six conservation organizations based in each country. These data were retrieved for Wildlife Conservation Society (https://www.wcs.org/about-us/offices), Fauna and Flora International (https://www.fauna-flora.org/countries), Conservation International (https://www.conservation.org/about/global-offices), Birdlife International (http://www.birdlife.org), International Union for Conservation of Nature (https://portals.iucn.org/library/sites/library/files/documents/2018-041-En.pdf) and World Wide Fund for Nature (https://wwf.panda.org/wwf_offices/wwf_offices_asia.cfm).

Geopolitical	Violence	Armed conflict data between 2010 and 2018 for each country. We adopted the Uppsala conflict database definition of armed conflict as "a contested incompatibility that concerns government and/or territory where the use of armed force between two parties, of which at least one is the government of a state, results in at least 25 battle-related deaths in one calendar year [3]." We extended the above definition to non-state based violence as well, e.g. between religious or ethnic groups. The geo-referenced data on occurrence of armed conflict were obtained from Uppsala Conflict Data Program (http://ucdp.uu.se).
Geopolitical	Worldwide Governance Indicators (WGI)	We used worldwide governance indicators (WGI) updated by World Bank. These indicators score countries on six measures of governance: voice and accountability; political stability and absence of violence; government effectiveness; regulatory quality; rule of law; and control of corruption. Each measure is scaled in the same way and we used the 2015 average across all six measures, obtained from https://datacatalog.worldbank.org/dataset/worldwide-governance-indicators.
Geopolitical	Gross domestic product (GDP) per capita	The GDP per capita, defined as the total market value of all final goods and services produced in a given country in a calendar year divided by the population, which is an indicator of a country's economic wealth per capita. GDP per capita (current US$) was obtained from World Bank Indicator portal [4].
Geopolitical	% Agriculture 2015 (% of land area)	The percentage of agricultural land to each country's land area was taken from World Bank statistical tables (https://data.worldbank.org). The last year available data was for 2015.
Geopolitical	Agricultural growth (% of land area)	The percentage of agricultural land growth in each country's land area was taken from World Bank statistical tables (https://data.worldbank.org), based on metrics available for 2010 and 2015.

References

1. UNEP-WCMC. *Protected Planet: The World Database on Protected Areas (WDPA), November 2020*. *UNEP-WCMC & IUCN* (2020).
2. Hickisch, R. *et al.* Effects of publication bias on conservation planning. *Conservation Biology* (2019).
3. Gleditsch, N. P., Wallensteen, P., Eriksson, M., Sollenberg, M. & Strand, H. Armed conflict 1946-2001: A new dataset. *Journal of peace research* **39**, 615–637 (2002).

4. World Bank. GDP per capita (current US$). https://data.worldbank.org/indicator/NY.GDP.PCAP.PP.CD/?most_recent_value_desc=true (2018).
